# First-line management of canine status epilepticus at home and in hospital-opportunities and limitations of the various administration routes of benzodiazepines

**DOI:** 10.1186/s12917-021-02805-0

**Published:** 2021-03-04

**Authors:** Marios Charalambous, Holger A. Volk, Luc Van Ham, Sofie F. M. Bhatti

**Affiliations:** 1 Small Animal Department, Faculty of Veterinary Medicine, Ghent University, 9820 Merelbeke, Belgium; 2 Department of Small Animal Medicine and Surgery, University of Veterinary Medicine Hannover, 30559 Hannover, Germany

**Keywords:** Dog, Emergency seizures, Epilepsy, Administration routes, Midazolam, Diazepam, Nasal

## Abstract

Status epilepticus (SE) or prolonged epileptic seizure activity is a common neurological emergency with a high mortality rate and, if left untreated, can lead to irreversible cerebral damage and systemic complications. Fast and effective first-line management is of paramount importance, particularly in the at-home management of seizures where drug administration routes are limited. Benzodiazepines (BZDs) have been exclusively used in veterinary medicine for decades as first-line drugs based on their high potency and rapid onset of action. Various administration routes exist in dogs, such as oral, intravenous, intramuscular, rectal, and intranasal, all with different advantages and limitations. Recently, intranasal drug delivery has become more popular due to its unique and favourable characteristics, providing potential advantages over other routes of drug administration in the management of canine SE. This narrative review provides an outline of the management of SE at home and in a hospital setting, discusses considerations and challenges of the various routes of BZD administration, and evaluates the impact of intranasal drug administration (nose-brain pathway) for controlling canine SE at home and within hospital settings.

## Background

Although most epileptic seizures are self-limiting and last for a few seconds or minutes (usually < 2–3 min), in some cases seizures can be prolonged leading to the development of status epilepticus (SE) [1]. SE is broadly defined clinically as seizures lasting > 5 min or multiple seizures with incomplete inter-seizure recovery and remains a common neurological emergency [1–3]. In self-limiting seizures, an array of processes lead to seizure termination including i) excitatory neurotransmitter (glutamate) and ATP depletion, ii) enhanced γ-aminobutyric acid (GABA)-induced inhibition, iii) adenosine release, iv) ionic (calcium, potassium) level alterations, and v) acidification of intra- and extracellular space [4]. Insufficiency of the seizure termination mechanisms and imbalance between excitatory and inhibitory activity within the forebrain’s neuronal network may lead to SE in both humans and animals [5, 6]. In addition, other mechanisms that promote seizure activity during SE include inflammatory processes (e.g. interleukins), enhanced pro-epileptogenic peptide expression (e.g. substance P), and blood-brain barrier (BBB) dysfunction [7–11].

SE can occur in dogs with idiopathic epilepsy (IE), structural epilepsy or reactive seizures [12–14]. In general, 0.5–2.6% of dogs are admitted to the emergency hospital for SE [15, 16]. Among the population of dogs presenting to hospital for seizures, 16.5% manifest SE [12] and the latter has been identified as the first clinical manifestation of an epileptic seizure disorder in 58% of dogs [16]. Studies showed that 32–40%, 27–59%, and 7–23% of dogs with structural epilepsy, IE, and reactive seizures, respectively, can present with SE [12–14, 16]. SE can lead to permanent brain damage (e.g. neuronal cell necrosis, network reorganization, gliosis) and severe systemic complications (e.g. cardiorespiratory collapse, shock, acidosis, electrolyte imbalances) [5]. The occurrence and severity of SE-induced complications are proportionally related to the duration of seizure activity [17–21]. With regard to the survival rates, an overall mortality rate of 25.3–38.5% among all dogs presented with SE has been reported [12, 16]. Therefore, clinicians or owners should quickly intervene to cease the continuous seizure activity either in hospital or at home, respectively [22–25]. Owners, in particular, have a substantial role in seizure control because appropriate administration of antiseizure drugs at home could prevent seizure progression to SE or reduce the risk of progression to more refractory stages. First-line management is of paramount importance and should include drugs with high potency and rapid onset of action. The aims of first-line management are i) cessation of seizures, ii) prevention of SE refractory phases, iii) prevention of complications, and iv) avoidance of adjunction of anaesthetic and non-anaesthetic antiseizure medications that could increase the risk of adverse effects [24]. Benzodiazepines (BZDs) have been exclusively used for decades in humans and animals as first-line antiseizure treatment due to their high potency and rapid onset of action [22–25].

The primary goals of this review are to i) provide an outline about the management of SE at home and in the hospital, with particular focus on first-line pharmacological intervention, ii) discuss the considerations and challenges of the various routes of BZD administration, iii) analyse and evaluate the recently introduced intranasal (IN) drug delivery method for controlling SE in dogs, and iv) provide guidance for primary and specialist clinicians regarding SE management within home and hospital settings.

### Therapeutic considerations in status epilepticus

**Fig. 1:**
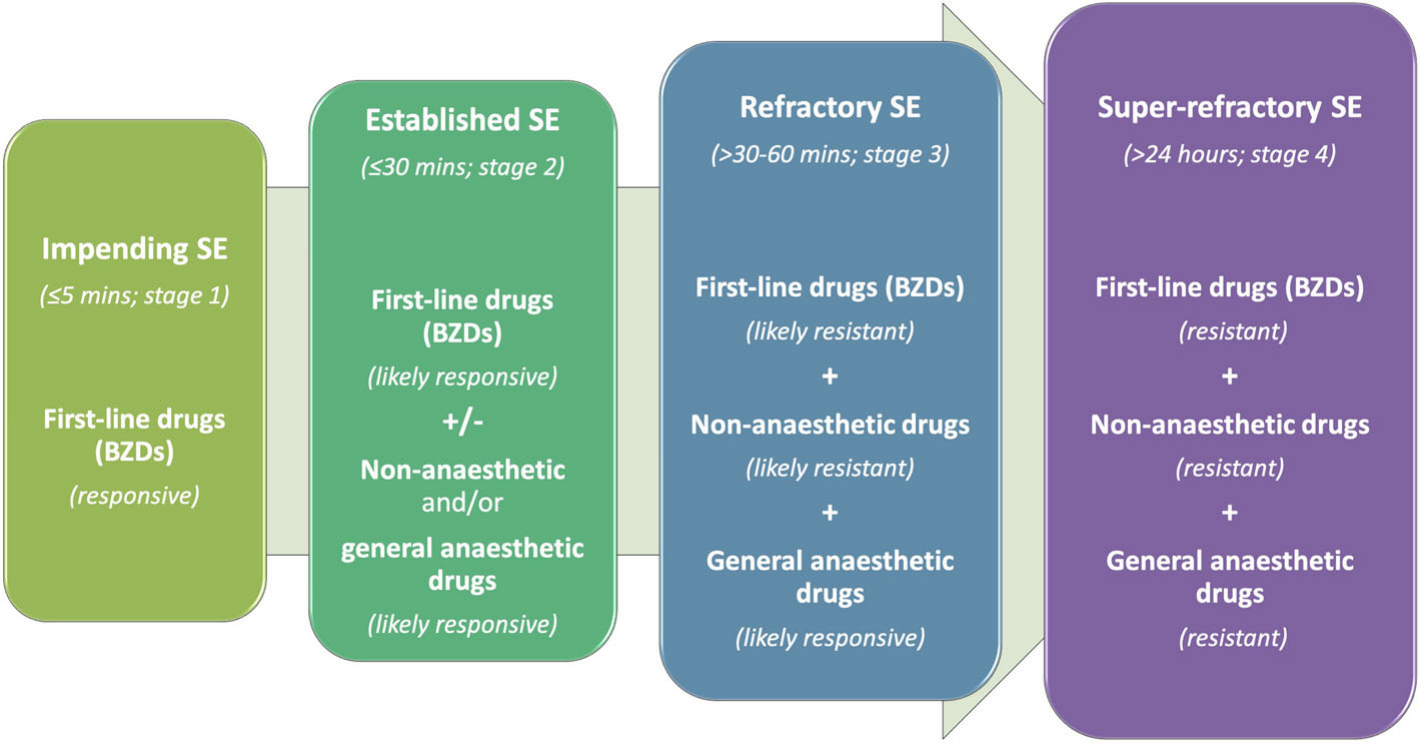
Stages of SE according to time and responsiveness to antiseizure medication. The more advanced the stage of SE is, the less responsive to antiseizure medication, and in particular first-line drugs (benzodiazepines), will be. Thus, in more advanced stages of SE, further antiseizure medication might be gradually added-on in order to control the epileptic seizures

SE therapy should incorporate a combination of medical interventions including antiseizure medication and measurements for treating seizure-related complications (e.g. intravenous fluid therapy for addressing in case of electrolyte imbalances) and underlying aetiological conditions (e.g. radiotherapy or surgical therapy in case of brain neoplasia, immunosuppressive therapy in case of immune-mediated meningoencephalitis) [5, 26]. Regarding seizure activity termination, SE may be subdivided into four different stages (Fig. 1), which differ in terms of sensitivity to the drugs used, treatment options as well as morbidity and mortality rates [17, 19, 20, 27–31]:

- Impending SE
  - Less than 5 min of continuous seizure activity.
  - Seizures are most likely responsive solely to first-line antiseizure therapy.
- Established SE
  - Less than 30 min of continuous seizure activity.
  - Seizures are still, but likely less, responsive to first-line antiseizure therapy.
  - Adjunctive non-anaesthetic (e.g. phenobarbital, levetiracetam) or general anaesthetic (e.g. propofol, ketamine, pentobarbital, etomidate, inhalation anaesthetics) antiseizure therapy might be needed.
- Refractory SE
  - Less than 30–60 min of continuous seizure activity.
  - Seizures are resistant to first-line and non-anaesthetic antiseizure therapy.
  - Adjunctive general anaesthetic antiseizure therapy is needed.
- Super-refractory
  - More than 24 h of continuous seizure activity or seizure recurrence after initiation of treatment with general anaesthetic antiseizure therapy.
  - Seizures are likely resistant to any antiseizure therapy.

**Fig. 2:**
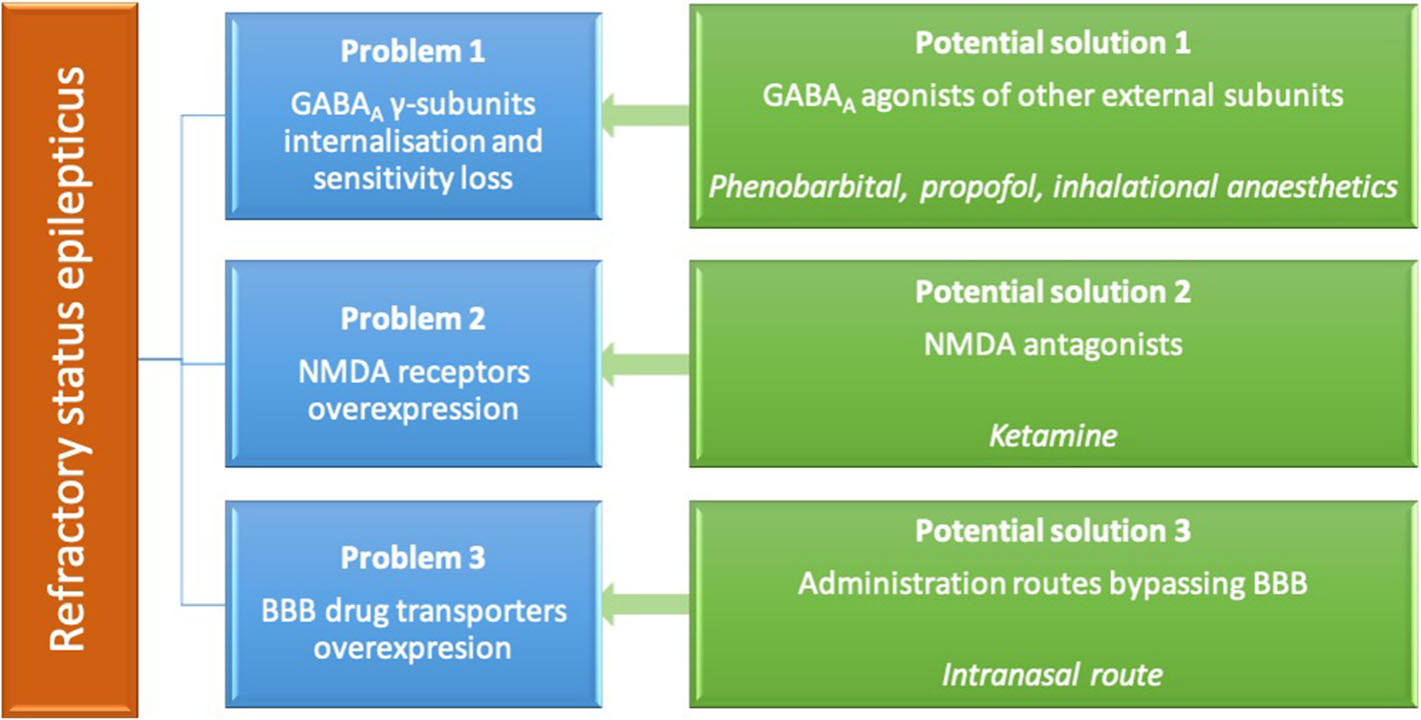
Summary of the main causes of pharmaco-resistance in refractory stages of SE and potential solutions to them

The reasons SE progresses towards more refractory stages over time are related to multiple processes that inhibit cessation of activity including mainly i) loss of GABA-induced inhibition, ii) upregulation of excitation induced by N-methyl-d-aspartate (NMDA) and α-amino-3-hydroxy-5-methyl-4-isoxazolepropionic acid (AMPA) receptors for glutamate, and iii) BBB transporters overexpression [6, 32]. GABAergic drugs (e.g. benzodiazepines (BDZs)) are particularly used in the management of SE [24, 25]. BZD’s effects derive from their action on pre- and postsynaptic GABA-ergic transmission; specifically, they bind on the γ-subunit of GABA_A_ receptors, enhancing the inhibitory effect of GABA, and result in an opening of chloride channels and influx of chloride within the neurons. This effect leads to hyperpolarisation of the cell membrane and inhibition of the transmission of nerve impulses [30, 33]. BZDs’ effectiveness, though, may gradually decrease with prolonged SE due to reduced synaptic targets (e.g. internalization of GABA_A_ receptors γ-subunits, alterations in GABA_A_ receptor trafficking and conversion of receptors subunits to less BZD-responsive) and changes in chloride homeostasis [34, 35]. Medications that act also on other external subunits (e.g. α, β) of GABA_A_ receptors (e.g. phenobarbital, propofol, inhalation anaesthetics) should be more efficient in cases of BZD-resistant SE [36–38]. In (super) refractory SE, resistance to most of GABA_A_-acting drugs may occur due to several factors including phosphorylation and internalization of the potassium-chloride transporter and increased concentration of intracellular chloride [39]. In addition, loss of AMPA receptors GluA_2_ subunit and overexpression of NMDA receptors occur, which promote glutamate-induced excitation [40, 41]; these changes lead to calcium accumulation within the cells and trigger apoptosis [41]. Glutamate receptor (NMDA) antagonists (e.g ketamine) may be beneficial particularly in refractory stages of SE and they may even help preventing resistance, if administered at early stages [6]. Overexpression and activation of NMDA receptors may also contribute towards calcineurin-induced internalization of the GABA_A_ receptors γ-subunits, leading further to BZD resistance [42, 43]. Therefore, NMDA receptor inhibitors may also have another benefit by means of enhancing BZDs potency as it was observed in animal models for SE [44–46]. Another process that occurs during SE is the overexpression of BBB drug transporters, which results in pharmaco-resistance [47]. A significant upregulation by 87–166% of endothelial P-glycoprotein (PGP; BBB drug transporter) was demonstrated in the canine brain following SE that leads to enhanced BBB efflux of antiseizure drugs and limited concentrations of drugs into the brain [48]. Lastly, after prolonged seizure activity, alterations in gene expression and associated protein production responsible for drug transporters as well as reorganization of synapses occur; all these processes contribute further to the drug resistance as it was shown in animal and human studies [17, 49, 50]. Therefore, the early application of drugs with different mechanisms of action (e.g. GABA_A_ agonists and NMDA antagonists) and through different routes (e.g. administration routes that might avoid BBB) with the aim to circumvent the mechanisms that sustain continuous seizure activity is fundamental for the management of SE (Fig. 2).

### Benzodiazepines

Diazepam (DZP), midazolam (MDZ), and lorazepam (LZP) are the main representatives of BZDs used as first-line treatment of SE [22–25]. In veterinary medicine, MDZ and DZP have been mainly used, although only DZP is currently licenced for small animals. In dogs, both DZP and MDZ can be effective for ceasing seizure activity, but this can depend on the administration route and dose [5, 22, 23, 25]; the recommended dose ranges for MDZ or DZP remain the same regardless the route of administration. Although not well-defined, it has been estimated that DZP should reach serum concentration of 0.15–0.5 μg/mL [33, 51–53] within 10–15 min in order to provide a clinically acceptable seizure control in canine SE and prevent progression to more refractory stages. For MDZ, these values have not been established for dogs, but it is speculated from human medicine that serum concentration of < 0.04 μg/mL might be adequate for seizure cessation [54]. In addition, MDZ might be potentially more potent than DZP because, in one canine study, MDZ manifested a higher suppressive effect on lidocaine-induced epileptic seizures compared to DZP [55]. In a human pharmacodynamic and encephalographic study, MDZ was found to be 5–6 times more potent than DZP [56]. In addition, MDZ has gained more popularity in the management of SE due to its safer drug profile, i.e. MDZ-induced brain and respiratory depression are less severe compared to DZP and LZP [30].

MDZ is a hydrophilic drug but converts to lipophilic at physiologic pH (e.g. tissues), which facilitates penetration of BBB [30, 56]. MDZ can be administered at a dose of 0.2–0.5 mg/kg IV, IN or intramuscular (IM) but has a short half-life (approximately 1 h in the dog), and thus, frequent administration or constant rate infusion (CRI) might be necessary for adequate seizure control [5, 25]. An IV CRI at the dose of 0.2–0.5 mg/kg/h might be advised to sustain good seizure control after the delivery of the initial MDZ bolus dose [5, 25]. The CRI dose is administered via an infusion pump and usually diluted in 0.9% saline or 5% dextrose solution, with the volume used being equal to the dog’s hourly maintenance fluid requirements [5, 25]. The dosage rate should be reduced by 50% every 6 h for at least two times before discontinuation of the drug [5, 25]. In humans, it has been shown that MDZ’s half-life increases after CRI [57].

DZP is a lipophilic drug, which can also penetrate the BBB [30, 56]. Because of its lipophilicity, it is prepared in propylene glycol, which can induce phlebitis and hypotension, especially when rapidly administered [5]. Therefore, central IV access may be needed for preventing phlebitis, although establishing such an access might be quite challenging and time-consuming during SE [5]. DZP can also adsorb to plastic and, therefore, should not be stored in plastic syringes or infusion lines for any length of time [58]. DZP may be administered as a bolus at the dose of 0.5–2.0 mg/kg IV, IN or rectally (R) [5, 25]. Repeat DZP bolus leads to accumulation and high concentrations of the drug in the central nervous system (CNS), cerebrospinal fluid (CSF), and serum; although this may result in prolonged antiseizure activity, it can also cause severe CNS depression and cardiorespiratory collapse [59]. Therefore, only 2–3 DZP boluses should be considered and, if unsuccessful, an IV CRI (at the dose of 0.1–0.5 mg/kg/h) or another antiseizure drug should be considered [5, 25]. Co-administration of levetiracetam enhances DZP’s antiseizure effect and, thus, DZP’s dose adjustment might be needed [60]; this phenomenon might also occur with the combination of levetiracetam and other BDZs [61].

### Administration routes

Various administration routes have been studied in human and veterinary medicine for managing SE either at home or in hospital. One of the main challenges is the at-home management of emergency seizures as therapeutic options and routes of administration are quite limited and restricted to non-IV routes of administration. Given the fact that SE management should be commenced at the earliest possible, there is an undoubted need for quickly effective routes of administration that can be applied in the out-of-hospital environment by owners or even clinicians within hospital settings (especially when IV line has not been yet established) [62]. Advantages and challenges of the various administration routes are discussed below and summarised in Table 1. Information regarding each BZD’s recommended dose and target serum concentration as well as reported serum concentrations, time to peak serum concentrations and time to seizure control achieved with each administration route in dogs is provided in Table 2.

**Table 1:** Advantages and limitations of benzodiazepines delivery routes in dogs

Administration route	Advantages	Limitations
**Intravenous**	Likely effective (clinical evidence) Likely rapid onset of action (clinical evidence) Precise control of the administered dose Avoidance of first-pass hepatic metabolism	Subject to blood-brain barrier Requirement for hospitalisation Requirement for medically-trained staff Hard to establish during seizures Not for at-home use
**Intramuscular**	Likely favourable pharmacokinetics Avoidance of first-pass hepatic metabolism	Subject to blood-brain barrier Requirement for training or medical staff Needle/syringe misuse by non-trained caregivers Less suitable for at-home use Soft tissue or nerve damage risk Infection risk Painful
**Transdermal**	Painless Easy to use Suitable for home No requirement for medical training Avoidance of first-pass hepatic metabolism	Subject to blood-brain barrier Slow release not suitable for emergency
**Buccal**	Painless Ease to administer Suitable for home No requirement for medical training Avoidance of first-pass hepatic metabolism	Subject to blood-brain barrier Potentially unfavourable pharmacokinetics Delivery of limited drug amount If swallowed, functions as oral Dog’s compliance is needed Incorrect administration during seizures
**Sublingual**	Similar to buccal	Similar to buccal
**Oral**	Painless Easy to use No requirement for medical training Suitable for home	Subject to blood-brain barrier Potentially unfavourable pharmacokinetics Slow absorption not suitable for emergency Potential for gastrointestinal degradation Subject to first-pass hepatic metabolism Dog’s compliance is needed
**Rectal**	Minimal pain/discomfort Relatively easy to use No requirement for medical training Suitable for home	Subject to blood-brain barrier Variability in effectiveness (clinical evidence) Variability in pharmacokinetics Partially subject to first-pass hepatic metabolism Likely slow onset of action Socially unacceptable
**Intranasal**	Likely effective (clinical evidence) Likely rapid onset of action (clinical evidence) Likely favourable pharmacokinetics Avoidance of first-pass hepatic metabolism Avoidance of blood-brain barrier No requirement for medical training Relatively easy to use Painless Suitable for home	Need for high concentration drug Potentially affected by mucosal factors Potentially affected by drug formulation Need for a veterinary nasal device

**Table 2:** Information regarding each benzodiazepine’s recommended dose and target serum concentration as well as reported serum concentrations, time to peak serum concentration and time to seizure control achieved with each administration route in dogs

	Midazolam	Diazepam
**Recommended dose**	0.2–0.5 mg/kg intravenous, intranasal or intramuscular	0.5–2.0 mg/kg intravenous, intranasal or rectal
**Recommended target serum concentration for seizure control** (pharmakokinetic studies)	< 0.04 μg/mL (value derived from humans)	0.15–0.5 μg/mL
**Serum concentration achieved with each administration route** (pharmakokinetic studies)	Intravenous	
	NA	NA
	Intranasal	
	0.21 ± 0.02 μg/mL (solution) or 0.45 ± 0.09 μg/mL (gel)	0.44 ± 0.04 μg/mL (solution) or 0.31 +/− 0.17 (solution/atomised formulation)
	Intramuscular	
	0.20 ± 0.06 μg/mL or 0.55 ± 0.12 μg/mL (solution)	NA
	Rectal	
	NA	0.5 μg/mL (solution) and or 0.01–0.1 μg/mL (suppository)
	Buccal	
	0.1–0.2 μg/mL (gel)	NA
	Sublingual	
	NA	NA
**Time to peak serum concentration achieved with each administration route** (pharmakokinetic studies)	Intravenous	
	NA	NA
	Intranasal	
	12 min (gel) or 17 min (solution)	4.5–8.0 min (solution/atomised formulation)
	Intramuscular	
	10–15 min (solution)	NA
	Rectal	
	NA	15 min (solution) or 80 min (suppository)
	Buccal	
	15 min (gel)	NA
	Sublingual	
	NA	NA
**Time to seizure control achieved with each administration route** (clinical studies)	Intravenous	
	1.0–4.5 min	NA
	Intranasal	
	0.5–1.6 min	NA
	Intramuscular	
	NA	NA
	Buccal	
	NA	NA
	Sublingual	
	NA	NA
	Rectal	
	NA	2.5 min

### Intravenous

IV administration of BZDs has an onset of action approximately within < 2–7 min, circumvents first-pass hepatic metabolism (i.e. i.e. liver-induced drug metabolism whereby the concentration of a drug might be substantially reduced) [30, 56], and is likely effective for ceasing SE in humans [63–71] and dogs [23, 25, 33, 72–74]. In humans, IV administration of BDZs has been considered the “gold standard” route as it can result in the highest drug efficacy and shorter seizure cessation time [75–77]; similar recommendations have been made in dogs [25, 78]. In clinical practice, however, delays in establishing IV access in a seizuring human [79–81] or dog [23, 72] can be significant and negatively affect IV drugs’ onset of action. Based on a systematic review/meta-analysis in humans, non-IV BDZs could be administered faster to patients and demonstrated superior efficacy and onset of action in terminating seizures compared to IV-BDZs [79]. In veterinary medicine, there are not enough clinical trials to allow the conduction of a systematic review/meta-analysis evaluating and comparing different BDZ or IV versus non-IV routes of administration, but it is likely that the experience and evidence derived from human medicine could be translated to veterinary medicine. Existing clinical data, though, already indicates that IV route, despite being effective, might not be the “gold standard” delivery method as it was widely speculated up to date. Specifically, based on a recent multicenter clinical trial in canine SE, IV administration of MDZ was inferior to IN with regard to median seizure cessation time (1 min for IV versus 0.5 min for IN), especially when the time needed to establish an IV catheter was considered (4.5 min for IV versus 1.6 min for IN) [23]. The main factors in clinical practice that pose significant difficulties in establishing or maintaining functional IV access and delays in IV drug administration and seizure cessation include i) convulsive seizure activity, ii) requirement of experienced medically-trained staff, iii) patient’s cardiovascular collapse, and iv) small or toy canine breeds [23, 68, 72, 79, 82, 83].

### Intramuscular

IM BZDs provide onset of action within 15 min after administration and have been suggested for at-home and in-hospital SE management in humans [64, 66, 84–88]. IM administration is not subject to first-pass hepatic metabolism and has been also shown that IM administration of MDZ can be as effective as IV-DZP for the management of human SE [86, 87]. In two double-blinded randomised controlled clinical studies in humans, IM-MDZ could be administered by trained medical staff quicker and easier than and was as effective as IV-LZP [64, 66]. Based on meta-analysis in humans, both IM and IN administration of BDZs have been shown to be two of the most effective and fastest methods for ceasing SE, especially in out-of-hospital settings [69, 89]. Clinical trials to evaluate IM-BZDs’ efficacy and safety have not been conducted in dogs, apart from pharmacokinetic studies [72, 73]. Specifically, after IM administration of MDZ solution (at the lowest clinically recommended dose of 0.2 mg/kg [72] or 0.5 mg/kg [73]), mean bioavailability was 50% [72] and > 90% [73]. The mean serum concentration was 0.20 ± 0.06 μg/mL [72] or 0.55 ± 0.12 μg/mL [73]. The maximum serum concentrations were achieved within 10–15 min [72, 73]. DZP is not advised to be given IM because of its erratic absorption [30].

In comparison to other non-IV routes, IM drug administration can be quite painful and pose risks [90], such as syringe/needle misuse, soft tissue or nerve injury, and administration in wrong sites or tissues, especially when administered by non-experienced, non-medically-trained individuals, such as dog owners.

### Transdermal

The transdermal drug administration is easily performed (no requirement for syringes or injections), not subject to first-pass hepatic metabolism, and could be a reasonable method for gradual and long-term delivery of drugs (lipophilic drugs with < 500 Da molecular weight, such as BZDs, can penetrate through the skin layers and reach the systemic circulation) [91–93]. However, before therapeutic levels of any drug appear to the systemic circulation, drug crossing and accumulation through the dermal layers is necessary [93, 94]; the latter depends on several factors such as pharmacological characteristics and delivery systems, skin thickness and barrier, and enzymes present in skin that degrade drugs [91–94]. Therefore, a rapid effect that is vital in emergency situations is unlikely in SE, even if permeation enhancers to increase drugs’ absorption are co-administered [91, 92]. The transdermal route for administering long-term antiseizure drugs, i.e. levetiracetam or phenobarbital, has been reported in epileptic cats [95–97] but there is a lack of evidence regarding transdermal BZDs for treating emergency seizures in dogs, likely due to the limitations discussed above.

### Buccal

Buccal-BZD might provide an alternative administration option in humans due to its relatively easy use (no requirement for syringes or injections) and the fact that it is socially acceptable (avoidance of rectal drug administration especially in public) [98]. Buccal MDZ has an onset of action within 5–10 min, avoids first-pass hepatic metabolism and has showed good efficacy and safety profile [98–104]. Based on a randomised controlled study, both buccal-MDZ and IV-DZP were successful in ceasing SE but IV-DZP had significantly better mean seizure cessation time (1.1 min) than buccal-MDZ (1.7 min); however, when the time to establish IV access was considered, buccal-MDZ demonstrated significantly shorter mean seizure cessation time (2.4 min) compared to IV (3 min), indicating that preparing the IV medication and introducing an IV line can delay the treatment [103]. According to a systematic review/meta-analysis, buccal-MDZ was more effective than R-DZP in ceasing seizures [69]. Buccal-MDZ, though, was not as effective and fast as IN-MDZ or IM-MDZ for terminating seizures, based on the conclusion of another systematic review/meta-analysis [89]. In dogs, only pharmacokinetic studies have been performed. One study showed that after buccal administration of various MDZ gel formulations (at the dose of 0.3 mg/kg), bioavailability ranged from 25 to 41% [105], mean serum concentrations ranged from 0.1–0.2 μg/mL and time to peak concentration was achieved within 15 min [105]. Another study showed a pH-dependent absorption of buccal-BZDs, with bioavailability ranging from 6.2–22.6% [106]. No clinical trials to support its efficacy in canine SE exist up to date.

Administering the correct dose via the buccal route poses limitations in humans (e.g. hypersalivation and risks of incomplete absorption and aspiration as well as need for patient’s cooperation that might not be realistic in cases of SE) [107]; these limitations might be higher in dogs, adding the risk of the owners being bitten or injured. Additionally, buccal route is beneficial only for small drug doses and volumes as some amount of the buccally administered drug can be swallowed; the latter can lead to decreased bioavailability and delayed time to peak concentration mainly due to the first-pass hepatic metabolism and gastrointestinal tract absorption time, respectively [108, 109].

### Sublingual

The sublingual route is another administration method within the oral cavity similar to buccal. The sublingual route provides a thinner and more permeable layer of absorption compared to buccal and, thus, could potentially provide a faster onset of action [110]. To benefit from this, it is essential that the drug should be administered in specific areas of the oral cavity, i.e. sublingual drugs are administered under the tongue, while buccal drugs at the caudal aspect of the oral cavity between the upper or lower molars and the cheek in humans. One of the main limitations in both routes is the necessity for cooperation of the patient for correct administration, which is quite challenging during SE and even more difficult or nearly impossible in dogs. The limitations mentioned in the buccal administration apply also in sublingual route. Absorption can also be very slow [111]. Therefore, sublingual and buccal drug delivery might not be ideal for humans and particularly dogs during seizures. This was also supported by one randomised controlled trial in 436 children showing that sublingual-LZP was less effective than R-DZP in managing seizures [112]. In dogs, no studies evaluating the sublingual BZDs administration have been performed.

### Oral

Oral is considered a practical and easy (no requirement for syringes or injections) route of drug administration [113], although it might not be feasible during SE. Certain oral drugs including BZDs and in particular MDZ display low or variable bioavailability in humans (approximately 53–97% and 15–40% for DZP and MDZ, respectively) as well as reduced efficacy and quite prolonged onset of action (approximately 15–60 and 10–45 min for DZP and MDZ, respectively) due to their slow absorption and enzymatic degradation in the gastrointestinal system (small intestine and stomach), and extensive first-pass hepatic metabolism [113–121]. In addition, oral BZDs cannot be administered in people with difficulty in swallowing or have severe CNS suppression [122], as it occurs in SE, and may lead to aspiration pneumonia, especially after administering oily solutions such as DZP. Similar limitations exist in dogs, including the risk of caregiver’s injury due to accidental dog bites, which impair the effect and use of oral BDZs in canine SE. BZDs’ mean availability after oral administration in dogs is 69% for MDZ [73] and > 70% for DZP [123]. Overall, oral BZDs are deemed inconvenient, risky as well as inadequate or ineffective in both human and canine SE.

### Rectal

Rectal administration of BZDs and in particular DZP has been well recommended and widely used as a relatively cheap and potentially effective managing option in human SE, with an onset of action within 10–15 min [124, 125]. Rectal drugs can be administered by non-medically trained individuals in contrast to IM and IV drug delivery routes [117]. Empty rectum provides a stable environment with low activity of degrading enzymes that favours absorption of drugs into the systemic circulation [117], but faecal material may impair drug absorption. R-DZP has been suggested as a non-IV method of treating SE in humans, especially within out-of-hospital settings [102, 126–128]. Based on a systematic review/meta-analysis the time periods from arrival in the hospital to drug administration and seizure cessation were shorter with IN, IM and buccal routes of MDZ administration compared to R-DZP [129]. Based on another meta-analysis, R-DZP was not considered as effective as other non-IV methods of MDZ administration and in particular IN- and IM-MDZ [89]. In a third systematic review, non-R BZDs routes of administration were suggested as better or preferred SE treatment options compared to R-DZP [130].

R-DZP in dogs has been widely recommended as a management option for SE in the absence of IV access. This recommendation has been mainly based on pharmacokinetic studies and one small-scale uncontrolled clinical trial [51–53, 131] with conflicting evidence. Specifically, after R administration of DZP (at the dose of 1 mg/kg as solution [52] or 2 mg/kg as solution [53] or 2 mg/kg as gel formulation/suppository [131]), mean bioavailability was reported to be 52% [52] or 7.4% [53] for the solution but was not detected for suppositories [131]. There was a notable variability in DZP serum concentrations among dogs but, in general, the mean serum concentration was approximately 0.5 μg/mL [52, 53] for the solution and ranged between 0.01–0.1 μg/mL for the suppository [131]. The maximum serum concentrations were achieved within 15 min [52, 53] for the solution and 80 min [131] for the suppository. In addition, one recent multicenter open-labelled controlled clinical study compared R-DZP to IN-MDZ and showed that R-DZP was successful in terminating SE in only 20% of the dogs (versus 70% in the IN-MDZ group) and was significantly inferior to IN-MDZ [22]. Hence, R-DZP, in particular suppositories, might provide variable results and potentially inadequate seizure control within the time frame needed for successful control of SE. Regarding R administration of MDZ, studies report erratic bioavailability and serum concentration ranging from undetectable to low [72, 73]. Therefore, MDZ is unlikely to be successful, but there are no clinical studies evaluating drug’s effect in SE.

R route of administration is generally not preferred by people due to cultural and social issues and the potential for discomfort and faecal or drug leakage out of the rectum [117]. Leakage of drugs together with other organic fluids can be an issue in dogs too, while application of rectal tubes might be difficult and performed incorrectly by the owners, especially during SE [22]. In addition, drugs can partially be subject to first-pass hepatic metabolism, which reduces their availability and increases their onset of action time [22, 117]. Given the fact that R-DZP in dogs with SE is relatively inconvenient and likely less successful compared to other routes [22, 131], the promising value of alternative delivery methods (i.e. IN) was highlighted in the recent years [22, 23].

### Intranasal

IN drug administration is a noninvasive method for delivering molecules and drugs aiming to act on local, systemic, and CNS level. IN delivery of BZDs offers multiple advantages because it i) requires minimum training and can be performed by non-medically trained individuals, ii) is easily executed, iii) carries minimal or no risk of injury for the owner, clinician or the dog (there were no reports of injury such as accidental bites of the personnel by the seizuring dog or trauma of the dog’s nostrils during IN device application and drug administration), and iv) is generally well accepted for use at home compared to other non-IV routes [22, 23, 122].

The IN route provides fast and efficient drug delivery to the brain. Specifically, human studies reported that IN-MDZ (at the minimum clinically recommended dose of 0.2 mg/kg) can reach the human brain and cease seizure activity within 2–5 min, as shown on electroencephalography [132]. In addition, IN-MDZ at the same dose can reach serum concetration of 0.1–0.18 μg/mL to achive sedation within 12 min after administration (minimum therapeutic concentration for sedation in adult humans is 0.04 μg/mL) [133–135]. It was suggested that the MDZ serum concentration needed to cease activity is even less compared to sedation in humans (< 0.04 μg/mL) [54]. IN-MDZ is also considered a good and successful alternative to other non-IV and IV routes of administration because its efficacy, safety and feasibility has been shown in multiple different species [22, 23, 122, 136–151]. Two human meta-analyses also strongly supported the effectiveness of IN-MDZ in SE [69, 89]. In one meta-analysis, IN-MDZ was found to terminate > 90% of seizures within 5–10 min and sustain seizure freedom for minimum an hour in 80% of people with SE [89].

In humans, both MDZ and DZP can be effective and potent via IN delivery [80, 152–154]. When compared, DZP is more lipophilic than MDZ, which can result in DZP’s better absorption by the nasal mucosa and potentially higher brain concentration [80, 152, 154]. However, DZP’s high lipophilicity also causes the drug to be rapidly redistributed into peripheral tissues which eventually results in DZP’s decreased concentration in the brain [80, 152]. MDZ demonstrates quicker rate of absorption by the nasal mucosa, but lower and more variable degree of absorption as well as shorter duration of action than DZP [80, 152–154]. However, MDZ’s higher potency and better safety profile compared to DZP [30, 55, 56] might make the drug a preferable choice in SE.

In veterinary medicine, pharmacokinetic studies showed that IN-MDZ [155, 156], IN-DZP [33, 157] and IN-flurazepam [156] are rapidly and efficiently absorbed by the nasal mucosa and can reach adequate therapeutic serum concentrations. Specifically, after IN administration of MDZ (lowest clinically recommended dose of 0.2 mg/kg) and DZP (lowest clinically recommended dose of 0.5 mg/kg), mean bioavailability was 52% (solution) [155] or 70.4% (gel formulation) [155] for MDZ and 80% (solution) [33] or 42% (solution/atomised formulation) [157] for DZP. The mean serum concentration was 0.21 ± 0.02 μg/mL (solution) [155] or 0.45 ± 0.09 μg/mL (gel formulation) [155] for MDZ and 0.44 ± 0.04 μg/mL (solution) [33] or 0.31 +/− 0.17 (solution/atomised formulation) [157] for DZP. The maximum serum concentrations were achieved within 17 min (solution) [155] or 12 min (gel formulation) [155] for MDZ and 4.5 min (solution) [33] or 8 min (solution/atomised formulation) [157] for DZP. Regarding results from veterinary clinical studies, two recent open-labelled randomised controlled clinical trials demonstrated that IN-MDZ was not only safe and superior to R-DZP but also superior to the “gold standard” IV route of MDZ administration, especially when the time to place an IV catheter was considered [22, 23].

An important consideration regarding IN administration of BZD is that drugs’ penetration into the brain and antiseizure effect can occur earlier than the time needed to reach maximum serum concentration [54, 155]. This can be supported by the fact that nasally administered drugs can follow both the blood systemic circulation and direct nerve pathways to reach the brain (as it will be thoroughly discussed later in the text); this may result in decreased drug concentration into the bloodstream, but successful penetration into the brain [90, 108, 158]. Therefore, estimating BZDs’ therapeutic serum concentration and bioavailability after IN administration might not be an accurate tool for estimating drugs’ efficacy, as it occurs with other administration routes.

### Nasal drug administration considerations

#### Anatomical considerations

The nasal cavity consists of two equal chambers (left and right), separated by the nasal septum, each of which has a vestibule (entrance of the nasal cavity) and main cavity. The nasal vestibule carries no cilia and is covered by stratified squamous epithelia [159]. The nasal vestibules’ blood perfusion is reduced compared to the main cavity, which results in insignificant drug absorption. The nasal sinuses can pose another potential area for drug absorption, but they are considered hard to reach due to their anatomical features (located into deeper and upper parts of nasal cavity with narrow passages and complex geometry) in both humans and dogs [90, 159–163]. The main nasal cavity consists of the respiratory and olfactory areas and is covered by highly vascularised mucus membranes, a fact that favours absorption into the systemic circulation. The respiratory region, in particular, consists of highly convoluted turbinates (conchae) and microvilli [164] that provide large surface-to-volume ratio and, hence, can benefit rapid drug absorption into the blood vessels.

#### Physiological considerations

**Fig. 3:**
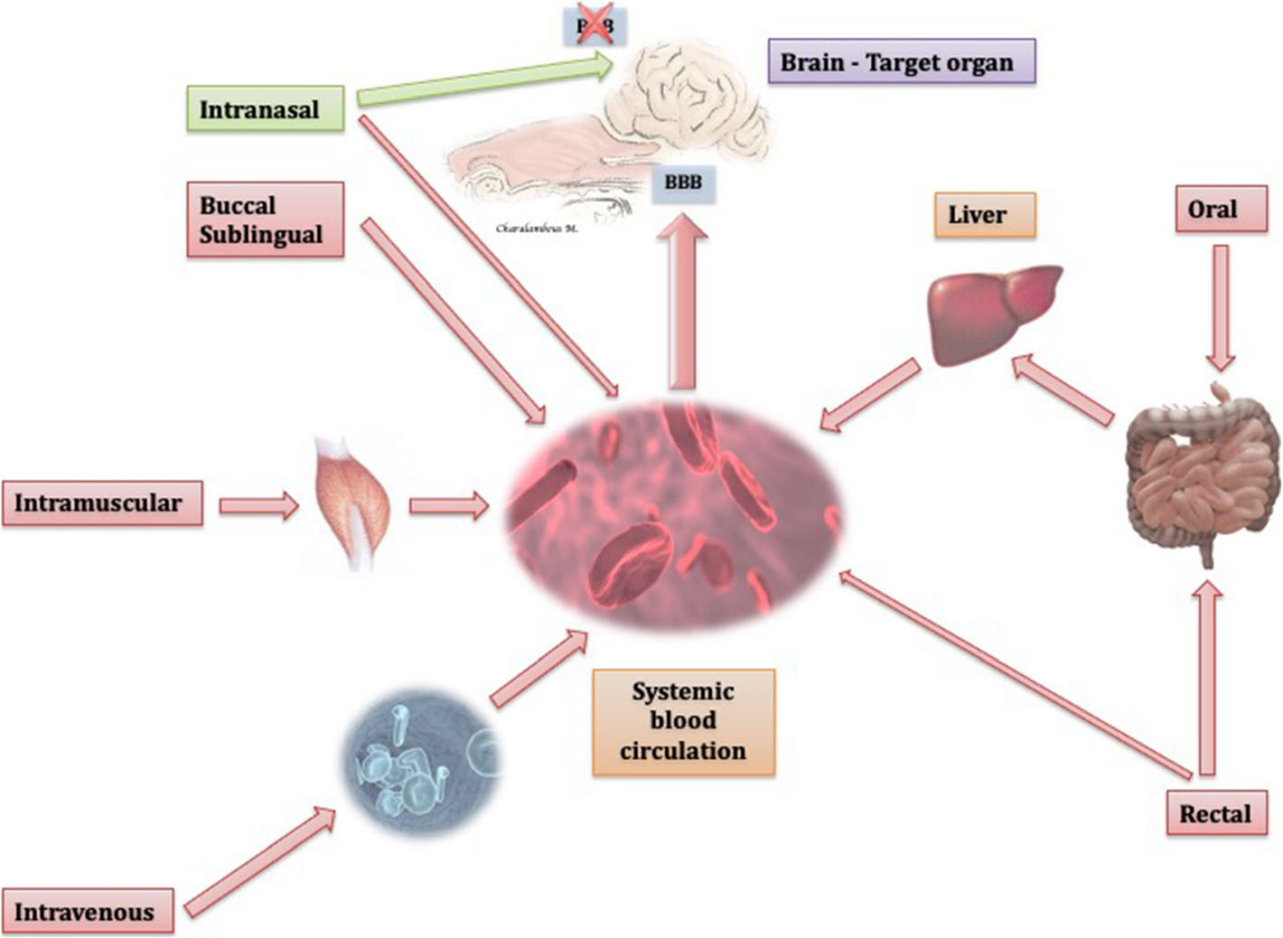
Schematic illustration of the different routes of drug administration’ pathways to the brain. The intranasal route is the only route that provides a direct pathway to the brain avoing the BBB (green arrow), along with an indirect pathway (red arrow). The remaining routes reach the brain indirectly (red arrows) via the systemic blood circulation passing via the BBB. Oral, in particular, and rectal route undergo first-pass hepatic metabolism, although rectally administered drugs could potentially avoid the first-pass metabolism, if they do not reach more cranial parts of the colon. Figures from authors’ personal record modified with microsoft power point

**Fig. 4:**
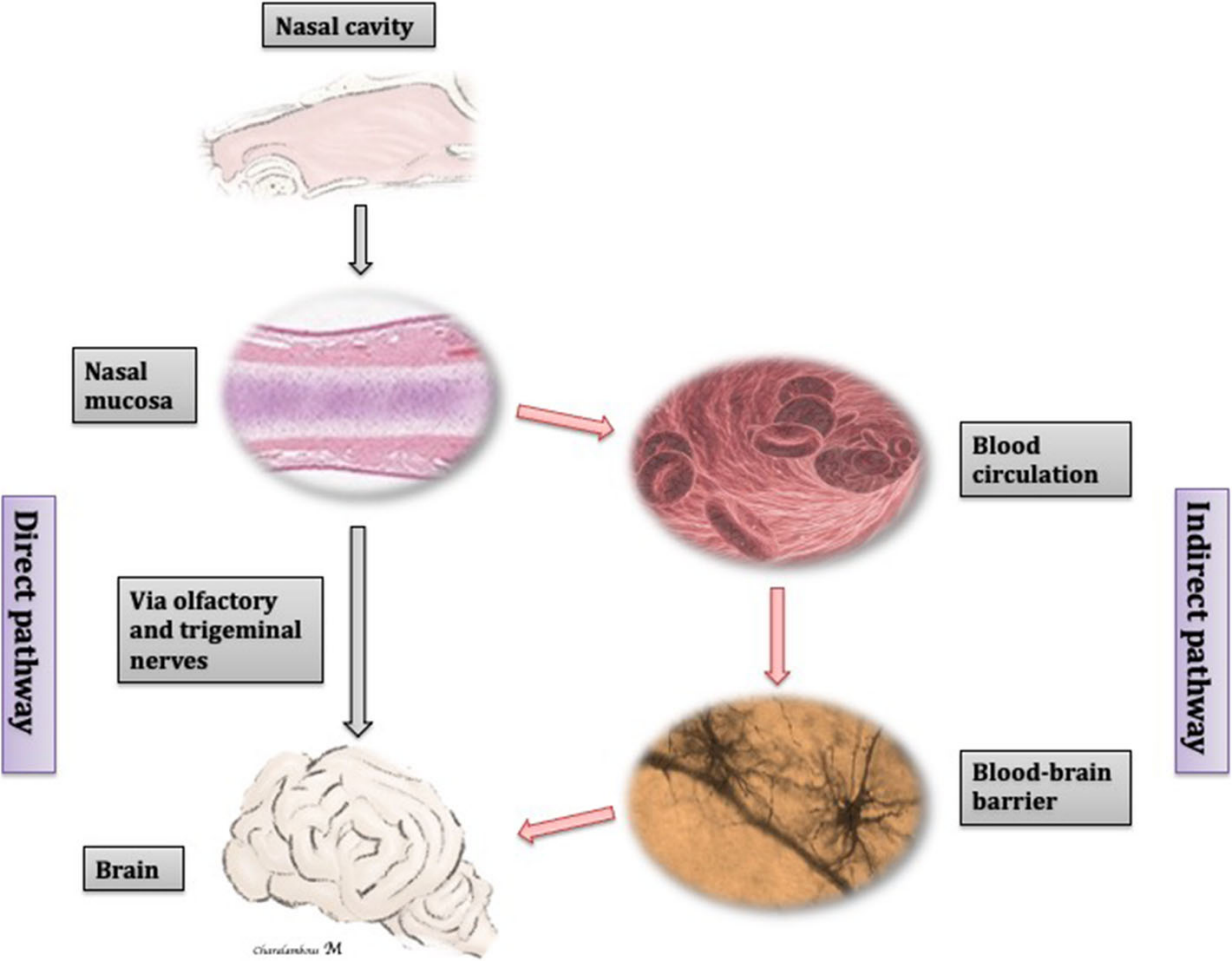
Schematic illustration of the indirect and direct (nose-brain) nasal drug delivery pathways. Drugs administered IN can penetrate directly into the brain through the olfactory and/or trigeminal nerve pathways or indirectly following absorption into the systemic blood circulation. Figures from authors’ personal record modified with microsoft power point

When compared to other administration routes, IN is the only route that can enter the brain via both the blood circulation (indirect pathway) and specific nerves (direct or nose-brain pathway), circumventing the BBB [90, 108, 158], as illustrated in Figs. 3 and 4.

##### Indirect nasal-brain drug delivery

The indirect pathway involves, firstly, a rapid drug absorption by the relatively large and highly-vascularised nasal epithelium and, secondly, delivery of the drug to the brain via the systemic circulation [90]. The less lipophilicity and higher molecular weight a drug exhibits, the less is absorbed by the nasal mucosa [109, 165, 166]. Lipophilic drugs with molecular weight < 1000 Da can be absorbed, but drugs with < 200 Da manifest the highest absorption [109, 165, 166]. Drugs are not subject to first-pass (presystemic) hepatic metabolism after absorption [90, 108, 158]. However, following absorption to the systemic circulation, IN drugs, similar to drugs administered via other routes, are subject to the systemic hepatic metabolism, renal function and plasma proteases, and they have to transverse the BBB for reaching the brain [90, 109, 167]. The BBB functions as a barrier, i.e., physical (intercellular tight junctions between endothelial cells and astrocyte end-feet), transport (multidrug transporters such as PGP), metabolic or enzymatic as well as immunological (perivascular mast cells, microglia and macrophages) [168, 169]. The physical barrier prevents molecules that are hydrophilic and/or have a high molecular weight (> 400–600 Da) to enter the brain [170]. Interestingly, more than 98% of the drugs cannot cross the BBB freely [171–174]. Modern drugs that target the brain are chemically modified to increase their stability and degree of BBB penetration [175]. BZDs are lipophilic drugs with molecular weight < 400 Da; therefore, not only can they be absorbed by the nasal mucosa to the systemic blood circulation, but the drugs can also penetrate the BBB and reach the brain [90, 166, 176].

##### Direct nasal-brain drug delivery

The direct pathway has gained attention in recent years as it offers a direct nose-brain axis for drug delivery via specific cranial nerves [164, 177–183]. Specifically, drugs gain access to the brain via the olfactory (nerve travels via the cribriform plate to provide special visceral afferent innervation to the olfactory mucosal epithelium) and trigeminal nerve (ophthalmic and maxillary branches travel via the cribriform plate to provide somatic afferent innervation to the respiratory mucosal epithelium) [178, 181, 184–186]. This nose-brain pathway is likely more advantageous for molecules that cannot enter the brain via other routes, as a result of their low systemic bioavailability (e.g. due to inadequate absorption into the systemic circulation or extensive metabolism and elimination) or inability to penetrate the BBB (e.g. hydrophilic or drugs with molecular weight > 400–600 Da) [90, 170]. There is accumulating evidence of IN administration of various hydrophilic and/or high molecular-weight molecules that reached rapid concentrations in the brain exceeding those that obtained after IV administration [158, 187, 188]; a fact that supports further the direct nose-to-brain pathway. In a study, it has been demonstrated that IN administration of drugs might lead to a drug CSF concentration that exceeds the blood plasma concentration [189–191] and can be identical to the direct intracerebroventricular administration [174]. In the olfactory region, in particular, there might be a possibility of another direct pathway, which could also contribute to the nose-brain pathway [173, 192, 193]. Specifically, the submucosal zone of the olfactory region is adjacent to the CSF flow paths of the olfactory bulb. Therefore, the IN drug could reach the CSF through the nasal epithelium and meninges that separate the submucosal space from the CSF [192].

Drugs following the direct pathway are transported via the nerves by various mechanisms including a) extracellular diffusion of the drug along the axonal myelin sheath and endoneurium of the nerves, b) extracellular convection of the drug following the fluid bulk flow through the perivascular zones of vessels that travel across the distal parts of the nerves and c) intracellular transport through the neuronal axons [185, 194, 195]. The extracellular convection of the drug (bulk flow) was suggested as the main mechanism of these nerve pathways, in particular for the olfactory nerve, that can be quick enough to cause the desired effect [182, 185, 195]. Final distribution of the drug from the point of entry into the brain, i.e. the olfactory bulb (drug entering through the nasal epithelium and olfactory nerve) and the brainstem (drugs entering through the trigeminal nerve), to other brain regions is likely performed via various transport mechanisms; these include intracellular (drug uptake and transfer via further connective neurons) and extracellular (drug distribution and transfer by convective bulk flow transport through the brain perivascular spaces or drug diffusion from the perivascular spaces into the brain parenchyma) [196–199].

##### Direct versus indirect pathway predominance in each nasal region

In humans, the respiratory and olfactory regions account for > 80–90% [164] and approximately 3–8% [164, 200] of the total nasal surface, respectively. The respiratory epithelium is considered more vascularised than the olfactory epithelium because one of its roles is to warm and humidify the inhaled air [201]. Therefore, the indirect pathway is likely favoured at the respiratory area, causing less amount of drug to become available for the direct (trigeminal nerve) pathway. In contrast, the olfactory area does not offer adequate highly vascularised surface [164, 200, 201] for the indirect pathway to occur and, thus, the direct (olfactory nerve) pathway is favoured. It might be possible that, due to the above anatomical reasons, trigeminal nerve might not be as significant as the olfactory nerve for transporting drugs into the human brain [90, 202]. On contrary, in dogs [162, 201] and rats [203], the respiratory and olfactory regions have almost equal distribution on the overall nasal cavity. Based on the fact that animals have remarkable larger olfactory area compared to humans [109, 162, 164, 203], it might be likely that there is similar drug distribution between direct and indirect methods of drug transport in each nasal area, although this assumption has not been proven yet.

#### Intranasal drug administration and pharmaco-resistance

A vital future consideration is a potential connection between IN route and pharmaco-resistance. As described earlier in the text, IN delivery of drugs may follow the direct or nose-brain pathway to enter the brain avoiding BBB vascular transporters, such as PGP. This can be quite advantageous for dogs with pharmaco-resistance, where there is impaired transfer of antiseizure drugs through the BBB due to overexpression of these transporters [48, 204, 205]. Therefore, it would be quite interesting to conduct future studies to assess the effect of IN delivery of various antiseizure drugs specifically in dogs with pharmaco-resistant epilepsy or refractory stages of SE.

### Intranasal drug administration potential challenges

#### Anatomical and physiological challenges of the nasal administration route

Even though IN route is promising for drug delivery into the brain, it can pose important challenges regarding drug absorption. First, hydrolytic enzymes (e.g. cytochrome P450 isoenzymes and aminopeptidases) are excreted by the nasal mucosa and can metabolise nasally administered drugs reducing their local or systemic bioavailability [200, 206]. Second, the mucociliary clearance (i.e. elimination of nasally entering substances by nasal mucosa) regulates the contact time of drugs with the nasal mucosa affecting the degree of their absorption [207]. Co-administration of mucoadhesion-enhancing agents could improve drugs’ contact time and absorption [90]. Third, there are epithelial transporters in nasal epithelium that can cause efflux of drugs from cells and reduce their absorption [200, 208–212]. Fourth, constriction or dilation of the nasal mucosa vessels can influence blood flow and, hence, drug absorption. Co-administering vasoconstriction agents (e.g. ephedrine or phenylephrine) can decrease drug nasal absorption [200, 213], while vasodilator agents (e.g. hydralazine) can enhance absorption [214, 215]. Fifth, nasal blood flow and drug absorption can be influenced by environmental factors such as nasal pathology, humidity, temperature, fear and stress [159, 216]. Lastly, the distribution of the IN drug can be potentially affected by anatomical features of specific canine breeds [217]. Precisely, in brachycephalic dogs, the conchae are hypertrophic, and the overall nasal cavity surface is decreased compared to dolichocephalic breeds [218, 219]; facts that could potentially limit the absorption and volume of nasally administered drugs. However, in two canine clinical studies [22, 23], various small, medium, and large breed as well as brachycephalic and dolichocephalic dogs were included, but no difference in the efficacy of IN-MDZ was detected among the dogs. Difficulties in applying the IN mucosal atomization device (MAD; nasal drug delivery device for MDZ) during SE were reported in < 24% of dogs [22, 23] and these were related to the initial unfamiliarity of personnel with the IN drug administration. Establishing IV access by placing an IV catheter [23] or using a syringe for R administration was perceived more difficult [22] during SE in dogs than applying the MAD at the entrance of the nasal cavity.

An ideal drug for IN administration should be characterised by adequate mucus solubility, ability for fast absorption, and rapid onset of action; highly concentrated solutions with small administration volume are also important because excess drug volume can flow out of the nasal cavity or drain into the oesophagus [59]. Combined with the above drug characteristics, attention should be given to the delivery device and the head position during administration as these factors can also affect drug’s distribution within the nasal cavity [220, 221]. Pump sprays are widely used in human medicine nowadays to deliver between 25 and 200 μL of drug volume per spray and they are relatively convenient and easy to use while allowing accurate dosage [222, 223]. Nasal drops [222] might be distributed over a larger area, although they might be cleared faster in comparison to sprays [224]. An essential limitation of both spray and, in particular, nasal drop systems is that they might require specific head positioning for correct administration, which can influence the drug’s distribution within the nasal cavity, absorption and therefore efficacy [222, 223]. In veterinary medicine, the IN drug delivery method has not yet well implemented or widely explored and there are no nasal devices specifically designed for dogs. In the two veterinary clinical trials evaluating the effect of IN-MDZ in dogs with SE [22, 23], a human device (i.e. MAD) was used for IN delivery and the majority of the dogs (70–76%) successfully responded. MAD functions like a syringe with a soft conical plug attached on it that converts the drug solution into an atomised mist. However, this device does not provide MDZ formulation already included in the device, requires time for preparation and drug administration and is not specifically adapted for the anatomical features of dogs. This can be problematic for small or brachycephalic breeds in which the correct application of the current human nasal devices might be challenging or even impossible. Dogs with SE may benefit from the design of a specific nasal device which would be adapted for small animals (e.g. thinner and more elongated device tip to adequately enter the nasal cavity and administer the drug into the whole nasal cavity) and contain drug solution ready for dosing and administration. Such a device might provide rapid and convenient delivery as well as enhanced efficacy of MDZ in dogs with SE.

## Conclusions

**Fig. 5:**
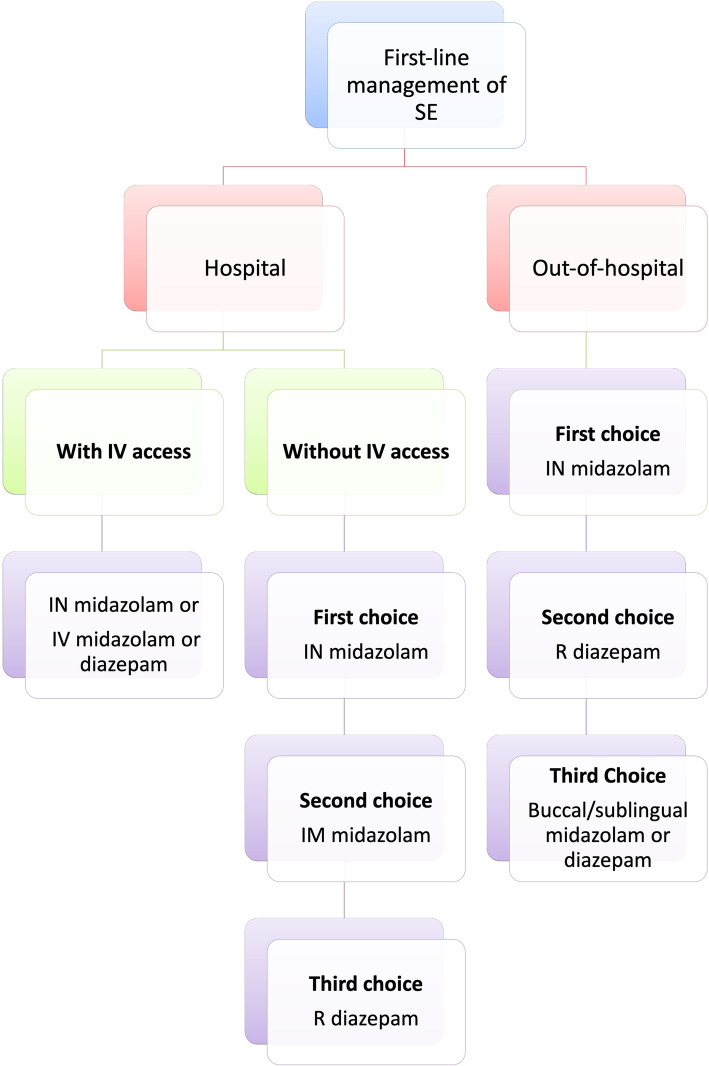
Cascade of choices for the first-line management of SE at home and in-hospital, with or without IV access

BDZs represent the first-line and widely-used treatment choice and still remain crucial for management of canine SE, despite their potential decline in effectiveness with more advanced SE stages. Multi-drug therapy, including drugs with different mechanisms of action, are also essential for a successful treatment. Evidence in dogs shows that efficacy and safety of non-IV routes of administration may be equal or even more effective to IV routes. This is more profound when the time to establish an IV catheter is considered. For at-home SE management, IN-MDZ is likely an effective and safe first-choice for terminating SE and well supported by clinical studies compared to other non-IV routes of administration. R-DZP is unlikely to be as effective as IN-MDZ to terminate SE, based on the current evidence. IM and buccal/sublingual administration routes might also be effective but there is currently insufficient to no clinical evidence supporting their efficacy and safety in canine SE, while their application at home by owners might be problematic. Regarding the in-hospital SE management, both IV- and IN-MDZ can be successful first choices but IN-MDZ can be advantageous particularly when IV access has not yet been established. Overall, based on the current evidence, IN-MDZ is recommended as a first-choice treatment in dogs with SE at home or in hospital and a proposed cascade is provided by the authors (Fig. 5). The IN pathway of drug delivery for SE provides several advantages as an administration method because it i) likely circumvents practical and efficacy-related issues associated with other IV and non-IV routes, ii) provides non-invasive and ease of administration, iv) offers capability for direct drug delivery into the brain, and v) provides enhanced drug bioavailability due to the high vascularisation of the nasal tissue, large surface available for drug absorption and avoidance of first-pass hepatic metabolism. Olfactory and trigeminal pathways might provide further advantages such as i) increased drug efficacy at lower dosages, ii) decreased risk for systemic adverse effects and iii) circumvention of BBB; the latter can be quite beneficial in pharmaco-resistant cases. These pathways are the only channels through which the brain is somewhat directly bridged to the external environment making the nose an effective “window to the brain”. A better understanding of the nasal anatomy and its limitations as well as formulation strategies can result in improved characteristics and efficacy of IN drugs.

## Data Availability

Not applicable.

## Abbreviations

BBB: Blood-brain barrier
BZD(s): Benzodiazepines
CNS: Cental nervous system
CRI: Constant rate infusion
CSF: Cerebrospinal fluid
DZP: Diazepam
GABA: Gabapentine
IE: Idioapthic epilepsy
IM: Intramuscular
IN: Intranasal
IV: Intravenous
LZP: Lorazepam
MDZ: Midazolam
NMDA: N-methyl-d-aspartate
PGP: P-glycoprotein
R: Rectal
SE: Status epilepticus
